# The impact of body weight on the development of peritoneal metastases in colorectal cancer patients: results from a nationwide cohort study

**DOI:** 10.1186/s12957-023-03204-5

**Published:** 2023-10-16

**Authors:** Vincent C. J. van de Vlasakker, Felice N. van Erning, Robin J. Lurvink, Ignace H. J. T. de Hingh, Simon W. Nienhuijs

**Affiliations:** 1 Department of Surgery, Catharina Cancer Institute, Catharina Hospital, PO Box 1350, Eindhoven, 5602 ZA the Netherlands; 2 Netherlands Comprehensive Cancer Organisation, Department of Research and Development, Utrecht, the Netherlands; 3 GROW-School for Oncology and Development Biology, Maastricht University, Maastricht, the Netherlands

**Keywords:** Colorectal neoplasms, Peritoneal metastases, Body mass index (BMI), Weight, Colorectal cancer, Risk factors

## Abstract

**Background:**

Obesity is a major global health problem and an important risk factor for colorectal cancer (CRC) is increased body weight. Obesity plays a role in the peritoneal dissemination of cancer; however, it is unclear whether this also applies for peritoneal dissemination of CRC. The purpose of this study was to provide insight in the role of obesity on the peritoneal dissemination of colorectal cancer.

**Methods:**

Of all patients diagnosed with CRC in the Netherlands in the first half of 2015, follow-up data was completed in 2019. Weight at time of primary diagnosis was categorized as underweight, normal weight, overweight, or obese. Logistic regression modelling was used to assess the association between weight and the presence of synchronous colorectal peritoneal metastases (CPM), and Cox regression modelling was used to assess the association between weight and metachronous CPM. Patient and tumor characteristics were taken into account. The analyses were adjusted for tumor stage, nodal stage, tumor location, and tumor histology.

**Results:**

In total, 6436 patients were included in this study. Two-hundred ninety-three (4.6%) patients presented with synchronous CPM at the time of primary diagnosis, while another 278 (5.1%) patients developed metachronous CPM after a median time of 16.5 months. Univariable and multivariable logistic regression modelling did not identify an effect of weight on the presence of synchronous CPM. Neither underweight (odds ratio [OR] 1.10, 95% *CI* 0.48–2.54), nor overweight (*OR* 0.96, 95% *CI* 0.71–1.29), or obesity (*OR* 0.84, 95% *CI* 0.56–1.26) was either positively or negatively associated with the presence of synchronous peritoneal metastases as compared to normal weight. Univariable and multivariable Cox regression modelling did not identify an effect of weight on the development of metachronous CPM. Neither underweight (*HR* 0.162, 95% *CI* 0.02–1.16), nor overweight (*HR* 1.07, 95% *CI* 0.82–1.39), or obesity (*HR* 1.02, 95% *CI* 0.73–1.16) was either positively or negatively associated with the presence of synchronous peritoneal metastases as compared to normal weight.

**Conclusion:**

CRC patients who are overweight or obese are not more at risk for the presence of synchronous CPM nor development of metachronous CPM than their normal-weight counterparts.

## Background

Colorectal cancer (CRC) constitutes a major global health burden, with nearly two million new cases every year [1]. In the Netherlands alone, there were over 11,000 newly diagnosed CRC patients in 2020 [2]. CRC disseminates often, and approximately 20% of newly diagnosed patients present with metastasized disease [2]. The peritoneum is the second most affected organ after the liver, with 5.7% of newly diagnosed CRC patients presenting with synchronous colorectal peritoneal metastases (CPM) [3].

This relatively large portion of CRC patients presenting with PM might in part be explained by the lack of clinical symptoms in early-stage CRC [3]. Obese patients in particular are at risk for underappreciation of symptoms, as many symptoms of advanced stage CRC, such as irregularity of bowel movements, can be caused by obesity itself [4, 5].

The role of obesity in CRC might be more extensive than just underappreciation of symptoms, as obesity is widely recognized to be a risk factor for the development of many types of cancer, amongst which CRC [6]. Moreover, obesity was shown to be a driver of the dissemination of cancer through the secretion of adipokines and pro-inflammatory cytokines [7]. Intra-abdominal cancers have a predilection to metastasize to peritoneal locations that are rich in adipocytes, as these adipocytes provide energy for tumor growth [8, 9]. Since obesity is associated with an increase in adipocyte size, it might be deduced that obese individuals have more energy available for tumor growth and are therefore more at risk for peritoneal metastases. However, whether this hypothesis for the role of obesity in peritoneal dissemination holds true for colorectal cancer remains to be elucidated.

Almost 40% of the worldwide population is currently overweight, and an additional 13% is living with obesity [1]. With an expected increase in these numbers, and the high prevalence of CRC and CPM worldwide, the role of obesity in the development and progression of CPM deserves to be investigated. The present population-based study aimed to provide insight into the effect of obesity on peritoneal dissemination of CRC.

## Methods

### Data source

Data from the Netherlands Cancer Registry (NCR) were used for this nationwide population-based cohort study. In this registry, all newly diagnosed malignancies in the Netherlands are registered by trained data managers who routinely extract data on patient, tumor, and treatment characteristics from hospital records. All patients diagnosed with CRC between January 1, 2015, and June 30, 2015, in the Netherlands were evaluated. In 2019, data managers from the NCR extracted follow-up data regarding local and systemic recurrences and their treatment from hospital records for all patients, while data on vital status were obtained through annual linkage to the municipal administrative database. Since all data were anonymized, no medical ethical approval was required for this study. The study was conducted in accordance with the Declaration of Helsinki and was approved by the scientific research board of the Netherlands Cancer Registry.

### Patient, tumor, and treatment characteristics

The anatomical sites of both primary tumors as well as of metastases were registered according to the International Classification of Disease-Oncology (ICD-O). Staging of disease was done according to the seventh edition of the tumor node metastasis (TNM) classification. Preferably, the pathological TNM (pTNM) stadium was used in the present study. If pTNM stadium was not available, clinical TNM (cTNM) stadium was used. Similarly, if patients were diagnosed with multiple primary tumors, the primary tumor that was initially diagnosed was used in the analyses. If the initial diagnosis of multiple primary tumors occurred simultaneously, the primary tumor with the highest TNM stage was included in the analyses.

The primary tumor location was categorized into three anatomical subsites according to the corresponding ICD-0 codes: (1) right-sided colon (C18.0, C18.2–18.4: cecum, ascending colon, hepatic flexure, transverse colon), (2) left-sided colon (C18.5–18.7: splenic flexure, descending colon, sigmoid), and (3) rectum (C19.9–20.9: rectosigmoid and rectum). Patients with a different primary tumor location (e.g., Appendix) were excluded.

The histology of the primary tumor was categorized into three histological subtypes: (1) adenocarcinoma (8000, 8010, 8020, 8140, 8144, 8210, 8211, 8220, 8255, 8261, 8262, 8263 and 8560), (2) mucinous adenocarcinoma (8480, 8481), and (3) signet ring carcinoma (8490). Patients with a different primary tumor histology (e.g., neuroendocrine tumor) were excluded.

Metastases were defined as peritoneal metastases (C16.0–16.9, C17.0–C17.9, C18.0–C18.9, C19.9, C20.9, C21.8, C23.9, C26.9, C48.0–C48.8, C49.4–C 49.5, C52.9, C53.9, C54.0–C54.9, C55.9, C56.9–C57.8, C66.9–C67.9, and C76.2) or as systemic metastases (any other metastatic location).

Metastases were defined in the NCR database as synchronous metastases if diagnosed < 90 days after diagnosis of the primary tumor and were defined as metachronous metastases if diagnosed ≥ 90 days after diagnosis of the primary tumor.

Curative treatment was defined as surgical treatment of primary CRC. Only patients without synchronous peritoneal metastases who underwent curative treatment were included in the subsequent analyses (i.e., identifying risk factors for the development of metachronous peritoneal metastases).

Weight and length, established at the time of the primary diagnosis, were used to calculate body mass index (BMI) in kg/m^2^, which was subsequently categorized into four categories: (1) underweight (BMI < 18.5), (2) normal weight (18.5 ≥ BMI < 25), (3) overweight (25 ≥ BMI < 30), and (4) obesity (BMI ≥ 30).

### Statistical analyses

Baseline characteristics were compared between patients amongst the four weight categories. Differences in continuous variables between patients amongst the four weight categories were compared using ANOVA tests and presented as mean ± standard deviation. Differences in categorical variables between patients amongst the four weight categories were compared using *χ*^2^ tests and presented as *n* (%). Missing data were excluded from the comparative analyses.

A multivariable binary logistic regression model was used to assess the association between weight and the presence of synchronous PM. The model was adjusted for patient and tumor characteristics with a *p*-value < 0.10 in univariable analyses.

A multivariable Cox regression model was used to assess the association between weight and the development of metachronous PM. The model was adjusted for patient and tumor characteristics with a *p*-value < 0.10 in univariable analyses. In order to prevent overfitting of the multivariable models, the number of variables was limited to ensure a minimum of 10 events per degree of freedom. Testing of multicollinearity was performed for each of the variables within the model. If a collinearity tolerance of < 0.25 occurred, or if a variance inflation factor (VIF) of > 3.0 occurred, corrections of the model would be performed. All tests were performed in a two-sided fashion, and a *p*-value of 0.05 was considered to be the upper limit for overall type 1 error, and thus, *p* < 0.05 indicated statistical significance. Statistical analyses were performed using SPSS, version 26.0 (IBM corporation Armonk, NY, USA).

## Results

**Fig. 1:**
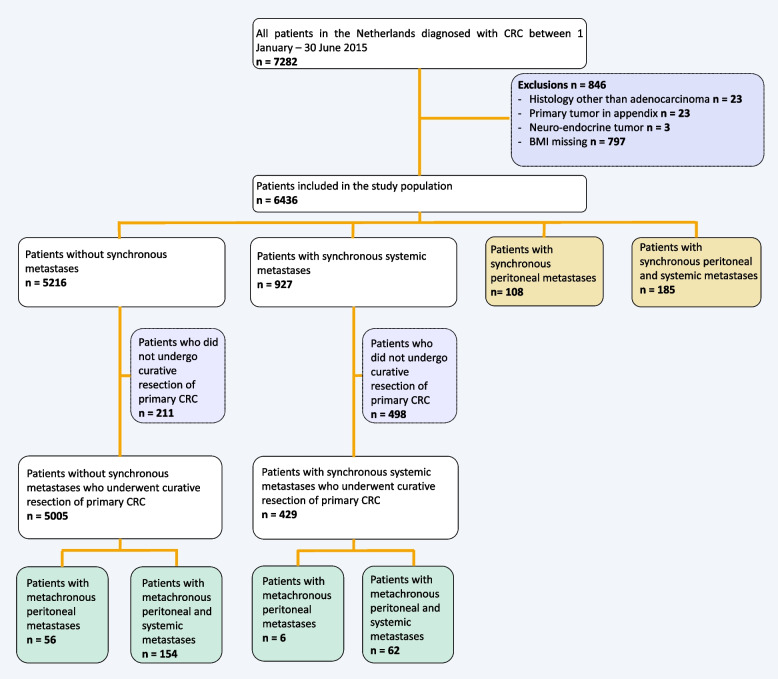
Flowchart of the study population with the incidence and onset of colorectal peritoneal metastases and systemic metastases

The final study population was comprised of 6436 patients (Fig. 1). In total, 293 (4.6%) patients were diagnosed with synchronous peritoneal metastases, of whom 108 were diagnosed as solitary PM and 185 as both synchronous peritoneal and systemic metastases. A total of 6143 patients were diagnosed without synchronous peritoneal metastases, and 88.5% (*n* = 5434) of them underwent curative treatment. Of this latter group, 278 (5.1%) patients were diagnosed with metachronous peritoneal metastases after a median time of 16.5 months (*IQR* 11.0–24.1).

Table 1 contains a detailed comparison of baseline characteristics between patients distributed over the weight categories. Patients with underweight (BMI < 20 kg/m^2^) were more often female, were more often diagnosed with a right-sided colon tumor, and diagnosed with synchronous peritoneal and systemic metastases in comparison to others (Table 1). Patients with obesity (BMI > 30 kg/m^2^) were more often men and were less often diagnosed with synchronous metastases (Table 1).

**Table 1:** Baseline characteristics of all patients per weight category

	***BMI* < 18.5 (*n* = 121) (2%)**	***BMI* 18.5–24.9 (*n* = 2448) (38%)**	***BMI* 25–29.9 (*n* = 2633) (41%)**	***BMI* > 30 (*n* = 1234) (19%)**	*p*-value^*^
**Age (years), mean (SD)**	68.9 ± 11.5	68.5 ± 11.0	68.4 ± 9.7	67.5 ± 8.8	0.012
**Sex, *n* (%)**					< 0.001
Male	34 (28.1)	1323 (54.0)	1709 (64.9)	682 (55.3)	
Female	87 (71.9)	1125 (46.0)	924 (35.1)	552 (44.7)	
**Tumor location, *n* (%)**					< 0.001
Right colon	51 (43.6)	770 (31.8)	779 (29.8)	377 (30.7)	
Left colon	32 (27.4)	845 (34.9)	942 (36.1)	507 (41.3)	
Rectum	34 (29.1)	805 (33.3)	891 (34.1)	344 (28.0)	
**Tumor differentiation, *n* (%)**					< 0.001
Good/moderate	77 (63.6)	1781 (72.8)	2058 (78.2)	968 (78.4)	
Poor/undifferentiated	15 (12.4)	225 (9.2)	210 (8.0)	107 (8.7)	
Missing	29 (24.0)	442 (18.1)	365 (13.9)	159 (12.9)	
**Tumor histology**					0.910
Adenocarcinoma	110 (90.9)	2238 (91.4)	2404 (91.3)	1113 (90.2)	
Mucinous adenocarcinoma	9 (7.4)	184 (7.5)	201 (7.6)	105 (8.5)	
Signet ring cell carcinoma	2 (1.7)	26 (1.1)	28 (1.1)	16 (1.3)	
**Synchronous PM, *n* (%)**					0.005
Yes	9 (7.4)	135 (5.5)	108 (4.1)	41 (3.3)	
No	112 (92.6)	2313 (94.5)	2525 (95.9)	1193 (96.7)	
**Synchronous systemic metastases, *n* (%)**					< 0.001
Yes	30 (24.8)	517 (21.1)	415 (15.8)	150 (12.2)	
No	91 (75.2)	1931 (78.9)	2218 (84.2)	1084 (87.8)	
**Tumor stage, *n* (%)**					< 0.001
T0–2	17 (15.3)	699 (30.2)	929 (35.3)	458 (37.1)	
T3	70 (63.1)	1191 (51.4)	1278 (48.5)	600 (48.6)	
T4	24 (21.6)	425 (17.4)	327 (12.4)	139 (11.3)	
Missing data	10 (8.3)	133 (5.4)	99 (3.8)	37 (3.0)	
**Nodal stage, *n* (%)**					0.238
N0	53 (43.8)	1019 (41.6)	1155 (43.9)	560 (45.4)	
N1	39 (32.2)	826 (33.7)	861 (32.7)	400 (32.4)	
N2	20 (16.5)	521 (21.3)	512 (19.4)	228 (18.5)	
Missing data	9 (7.4)	82 (3.3)	105 (4.0)	46 (3.7)	

*BMI* Body mass index (in kg/m^2^), *n* Number, *SD* Standard deviation, *PM* Peritoneal metastases

^*^Missing data were excluded from the comparative analyses

### The presence and development of peritoneal metastases

Synchronous peritoneal metastases were diagnosed in 4.9% of CRC patients (Table 1).

Amongst patients without synchronous peritoneal metastases, who underwent curative treatment (*n* = 5434), 278 patients (5.1%) were diagnosed with metachronous peritoneal metastases after a median time of 16.5 months (*IQR*: 11.0–24.1 months). The 1- and 3-year cumulative incidence of metachronous peritoneal metastases were 1.8% and 5.0%, respectively.

Patients with normal weight and patients with overweight were comparable in regard to the diagnosis frequency of synchronous and metachronous PM (normal weight: 5.5% vs 5.0%, respectively; overweight 4.1% vs 5.2%, respectively).

### The association between weight and the presence of synchronous peritoneal metastases

Univariable logistic regression analyses are presented in Table 4 in the Appendix. Multivariable logistic regression analyses (Nagelkerke *R*^2^ = 0.352) are presented in Table 2. Neither underweight (*OR* 1.10, 95% *CI* 0.48–2.54), nor overweight (*OR* 0.96, 95% *CI* 0.71–1.29), or obesity (*OR* 0.84, 95% *CI* 0.56–1.26) was either positively or negatively associated with the presence of synchronous peritoneal metastases as compared to normal weight. Variables positively associated with the presence of synchronous peritoneal metastases were tumor stage, nodal stage, tumor histology, and the presence of synchronous systemic metastases (Table 2). The rectum as primary tumor location was found to be negatively associated with the presence of synchronous peritoneal metastases (Table 2). No violations of the multicollinearity assumptions occurred.

**Table 2:** Multivariable logistic regression analyses for the presence of synchronous peritoneal metastases

**Variable**	*Crude rate synchronous PM*	**Multivariable logistic regression analysis**		
	*n (%)*	*OR*	*95% CI*	*p*-value^*^
**Weight category**				
1. (BMI < 18.5); underweight	9 (7.4)	1.099	0.477–2.535	0.824
2. (BMI 18.5–25); normal weight	135 (5.5)	Ref	Ref	Ref
3. (BMI 25–30); overweight	108 (4.1)	0.955	0.707–1.288	0.761
4. (BMI ≥ 30); obesity	41 (3.3)	0.840	0.558–1.263	0.401
**Primary tumor location**				
Right colon	137 (6.9)	1.02	0.759–1.368	0.898
Left colon	116 (5.0)	Ref	Ref	Ref
Rectum	26 (1.3)	0.291	0.182–0.464	** < 0.001**
**Tumor differentiation**				
Good/moderate	124 (2.5)	Ref	Ref	Ref
Poor/undifferentiated	49 (8.8)	1.223	0.814–1.838	0.332
**Tumor stage**				
T0–2	6 (0.3)	Ref	Ref	Ref
T3	68 (2.2)	4.203	1.780–9.901	**0.001**
T4	146 (16.0)	19.821	8.408–46.72	** < 0.001**
**Nodal stage**				
N0	55 (2.0)	Ref	Ref	Ref
N1	75 (3.5)	1.185	0.796–1.765	0.402
N2	120 (9.4)	2.145	1.433–3.212	** < 0.001**
**Tumor histology**				
Adenocarcinoma	224 (3.8)	Ref	Ref	Ref
Mucinous adenocarcinoma	48 (9.6)	2.337	1.525–3.579	** < 0.001**
Signet ring cell carcinoma	21 (29.2)	4.930	2.481–9.796	** < 0.001**
**Synchronous systemic metastasis**				
No	108 (2.0)	Ref	Ref	Ref
Yes	185 (16.6)	4.726	3.432–6.508	** < 0.001**

*PM* Peritoneal metastases, *n* Number, *OR* Odds ratio, *CI* Confidence interval, *BMI* Body mass index (in kg/m^2^)

^*^Missing data were excluded from the regression model

### The association between weight and the development of metachronous peritoneal metastases

Univariable Cox regression analyses are presented in Table 5 in the Appendix. Multivariable Cox regression analyses are presented in Table 3. Neither underweight (*HR* 0.162, 95% *CI* 0.02–1.16), nor overweight (*HR* 1.07, 95% *CI* 0.82–1.39), or obesity (*HR* 1.02, 95% *CI* 0.73–1.16) was either positively or negatively associated with the development of metachronous peritoneal metastases as compared to normal weight (Table 3). Patients who were diagnosed with a mucinous adenocarcinomas or signet ring cell tumors were more likely to develop metachronous PM. Other variables positively associated with the development of metachronous peritoneal metastases were a more advanced tumor stage (i.e., T3 or T4 tumor stage), a more advanced nodal stage (i.e., N1 or N2 nodal stage), and the presence of synchronous systemic metastases (Table 2). No violations of the multicollinearity assumptions occurred.

**Table 3:** Multivariable Cox regression analyses for the development of metachronous peritoneal metastases after curative primary tumor resection

Variable	Crude rate metachronous PM after median FU of 16.5 months	Multivariable Cox regression analysis^*^		
	*n (%)*	*HR*	*95% CI*	*p-value*
**Weight category**				
1. (BMI < 18.5); underweight	1 (1.1)	0.162	0.023–1.159	0.125
2. (BMI 18.5–25); normal weight	107 (5.4)	Ref	Ref	Ref
3. (BMI 25–30); overweight	117 (5.2)	1.065	0.819–1.386	0.638
4. (BMI ≥ 30); obesity	53 (4.8)	1.017	0.730–1.159	0.919
**Tumor differentiation**				
Good/moderate	206 (4.7)	Ref	Ref	Ref
Poor/undifferentiated	38 (8.7)	1.232	0.862–1.761	0.252
**Tumor stage**				
T0–2	20 (1.0)	Ref	Ref	Ref
T3	152 (5.5)	4.003	2.456–6.525	**< 0.001**
T4	106 (17.0)	11.447	6.844–19.14	**< 0.001**
**Nodal stage**				
N0	51 (2.0)	Ref	Ref	Ref
N1	118 (6.5)	2.038	1.456–2.852	**< 0.001**
N2	107 (11.0)	2.836	1.898–4.045	**< 0.001**
**Tumor histology**				
Adenocarcinoma	233 (4.7)	Ref	Ref	Ref
Mucinous adenocarcinoma	34 (8.2)	1.979	1.313–2.983	**0.001**
Signet ring cell carcinoma	11 (24.4)	4.455	2.247–8.831	**< 0.001**
**Synchronous systemic metastasis**				
Yes	68 (15.9)	3.045	2.279–4.068	**< 0.001**
No	210 (4.2)	Ref	Ref	Ref

*PM* Peritoneal metastases, *n* Number, *HR* Hazard ratio, *CI* Confidence interval, *BMI* Body mass index (in kg/m^2^)

^*^Missing data were excluded from the regression model

## Discussion

The present study found that weight was not associated with the presence of synchronous colorectal peritoneal metastases, nor was it associated with the development of metachronous colorectal peritoneal metastases. This is an interesting finding, since previous research has shown that 11% of all primary colorectal cancer (CRC) cases in Europe can be attributed to an increased weight status (i.e. a BMI within the overweight or obese categories) [10].

Intra-abdominal cancers, such as colorectal cancer, have a preference to disseminate to the omentum, a peritoneal organ that consists largely of adipocytes, suggesting a role of adipocytes in the dissemination of cancer [11]. Mouse models of ovarian cancer have shown that omental adipocytes promote migration and invasion of ovarian cancer cells, and that ovarian cancer cells utilize the readily available fatty acids as a source for growth [8]. Obesity promotes peritoneal dissemination of ovarian cancer, as adipocytes and fatty acids are more abundantly present in the abdomen of obese individuals [9]. Moreover, obesity stimulates the secretion of adipokines and pro-inflammatory cytokines, thus priming the peritoneal tumor environment further for metastasis growth [9].

We hypothesized that in CRC, similar effects might occur and thus expected a higher incidence of synchronous and metachronous peritoneal metastases in patients who were overweight or obese. However, in this prospective nationwide cohort, no effect of weight on the presence of synchronous peritoneal metastases, nor on the development of metachronous peritoneal metastases, was observed. This suggests that, unlike ovarian cancer cells, colorectal cancer cells might be relatively insensitive to obesity-related priming of the microenvironment and dissemination thereto.

However, the absence of a relationship between increased weight and the presence of synchronous CPM or development of metachronous CPM might in part reflect the limitations of weight as an approximation of metabolic health. The dysfunctional and pro-inflammatory processes that promote cancer and that are often attributed to obesity are a result of unhealthy metabolic processes, rather than of weight itself [9, 12]. This could have attenuated the results of the present study, as no distinction could be made between metabolically healthy obese individuals and metabolically unhealthy normal weight individuals. Indeed, in a previous study that investigated the role of obesity on the development of peritoneal metastases, it was reported that increased BMI has a protective effect on peritoneal seeding. Simultaneously, it was reported that increased visceral adipose tissue ratios, a more adequate approximation for metabolic health, exerted a hazardous effect [13]. Their contradictory findings emphasize the limitations of the use of weight in research.

In addition to weight being an imperfect approximation of metabolic health, other limitations to the present study were caused by the nature of these nationwide data and might have contributed to the rejection of our research hypothesis. The NCR only registers a certain set of variables, which does not include the peritoneal cancer index (PCI) score. If obesity would induce intra-abdominal cancer growth, rather than its dissemination, this could have resulted in a higher PCI score in individuals who were obese. However, this could not be assessed due to limitations of the data, and future research and registries should focus on this issue. Additionally, despite being a nationwide cohort, the number of patients with peritoneal metastases was limited, thereby restricting the power of the study and thus decreasing the likelihood of accepting our research hypothesis. Furthermore, due to the timing of the baseline measurements (at the time of the primary cancer diagnosis), weight, and thus BMI, might have been affected by the cancer even prior to the diagnosis. Thereby, patients who experienced cancer-associated weight loss might have been categorized into a lower BMI category. Lastly, due to the retrospective nature of the data, it was unknown whether patients had undergone true curative surgery for their primary tumor and their synchronous systemic metastases or merely a palliative resection of the primary tumor. As some of these patients might not have been cured, they could have had a higher rate of developing peritoneal metastases, thereby causing an overestimation of the incidence of metachronous peritoneal metastases. Further research is needed to resolve and overcome these limitations and to provide robust evidence on this topic.

## Conclusion

This study found no relationship between increased weight and the presence of synchronous CPM, nor between increased weight and the development of metachronous CPM. Thus, CRC patients with overweight and obesity are not at a higher risk for CPM, despite possibly delayed presentation of the primary tumor, more abundantly available energy sources within the peritoneum, and dysfunctional metabolic processes.

## Data Availability

Data will be made available by corresponding author upon reasonable request.

## Appendix

**Table 4:** Univariable logistic regression analyses for the presence of synchronous peritoneal metastases

**Variable**	*Synchronous PM*	**Univariable logistic regression analysis**		
	*n (%)*	*OR*	*95% CI*	*p-value*
**Age** (years)	293 (4.6)	0.999	0.987–1.10	0.819
**Sex**				0.750
Male	168 (4.5)	Ref	Ref	
Female	125 (4.7)	1.039	0.820–1.317	
**Primary tumor location**				< 0.001
Right colon	137 (6.9)	1.42	1.01–1.83	
Left colon	116 (5.0)	Ref	Ref	
Rectum	26 (1.3)	0.24	0.16–0.37	
**Tumor differentiation**				< 0.001
Good/moderate	124 (2.5)	Ref	Ref	
Poor/undifferentiated	49 (8.8)	3.703	2.627–5.128	
**Tumor stage**				< 0.001
T0–2	6 (0.3)	Ref	Ref	
T3	68 (2.2)	7.739	3.352–17.864	
T4	146 (16.0)	66.355	29.207–150,749	
**Nodal stage**				< 0.001
N0	55 (2.0)	Ref	Ref	
N1	75 (3.5)	1.816	1.277–2.584	
N2	120 (9.4)	5.134	3.704–7.116	
**Tumor histology**				< 0.001
Adenocarcinoma	224 (3.8)	Ref	Ref	
Mucinous adenocarcinoma	48 (9.6)	2.680	1.934–3.714	
Signet ring cell carcinoma	21 (29.2)	10.369	6.131–17.537	
**Weight category**				0.005
1. (BMI < 18.5); underweight	9 (7.4)	1.377	0.683–2.775	
2. (BMI 18.5–25); normal weight	135 (5.5)	Ref	Ref	
3. (BMI 25–30); overweight	108 (4.1)	0.733	0.565–0.950	
4. (BMI > 30); obesity	41 (3.3)	0.589	0.412–0.841	
**Synchronous systemic metastasis**				< 0.001
No	108 (2.0)	Ref	Ref	
Yes	185 (16.6)	9.638	7.526–12.344	

*PM* Peritoneal metastases, *n* Number, *OR* Odds ratio, *CI* Confidence interval, *BMI* Body mass index (in kg/m^2^)

**Table 5:** Univariable cox regression analyses for the development of metachronous peritoneal metastases after curative primary tumor resection

**Variable**	*metachronous PM*	**Univariable Cox regression analysis**		
	*n (%)*	*HR*	*95% CI*	*p-value*
**Age** (years)	278 (5.1)	0.991	0.979–1.003	0.146
**Sex**				0.450
Male	156 (4.9)	Ref	Ref	
Female	122 (5.4)	1.096	0.864–1.388	
**Primary tumor location**				0.189
Right colon	107 (6.4)	1.286	0.981–1.685	
Left colon	106 (5.2)	Ref	Ref	
Rectum	65 (3.8)	1.155	0.846–1.576	
**Tumor differentiation**				**< 0.001**
Good/moderate	206 (4.7)	Ref	Ref	
Poor/undifferentiated	38 (8.7)	2.173	1.537–3.071	
**Tumor stage**				**< 0.001**
T0–2	20 (1.0)	Ref	Ref	
T3	152 (5.5)	5.852	3.674–9.328	
T4	106 (17.0)	18.822	11.672–30.354	
**Nodal stage**				**< 0.001**
N0	51 (2.0)	Ref	Ref	
N1	118 (6.5)	3.339	2.405–4.638	
N2	107 (11.0)	6.6412	4.593–8.952	
**Tumor histology**				**< 0.001**
Adenocarcinoma	233 (4.7)	Ref	Ref	
Mucinous adenocarcinoma	34 (8.2)	1.786	1.247–2.560	
Signet ring cell carcinoma	11 (24.4)	6.277	3.428–11.494	
**Weight category**				0.376
1. (BMI < 18.5); underweight	1 (1.1)	0.215	0.030–1.537	
2. (BMI 18.5–25); normal weight	107 (5.4)	Ref	Ref	
3. (BMI 25–30); overweight	117 (5.2)	0.913	0.703–1.187	
4. (BMI > 30); obesity	53 (4.8)	0.857	0.617–1.191	
**Synchronous systemic metastasis**				**< 0.001**
Yes	68 (15.9)	5.374	4.083–7.073	
No	210 (4.2)	Ref	Ref	

*PM* Peritoneal metastases, *n* Number, *HR* Hazard ratio, *CI* Confidence interval, *BMI* Body mass index (in kg/m^2^)

## Abbreviations

BMI: Body mass index
CI: Confidence interval
CPM: Colorectal peritoneal metastases
CRC: Colorectal cancer
HR: Hazard ratio
ICD-O: International Classification of Disease-Oncology
NCR: Netherlands Cancer Registry
OR: Odds ratio
PCI: Peritoneal cancer index
PM: Peritoneal metastases
SD: Standard deviation
TNM: Tumor node metastasis
